# Ethnicity and prediction of cardiovascular disease: performance of QRISK2 and Framingham scores in a UK tri-ethnic prospective cohort study (SABRE—Southall And Brent REvisited)

**DOI:** 10.1136/heartjnl-2013-304474

**Published:** 2013-11-01

**Authors:** Therese Tillin, Alun D Hughes, Peter Whincup, Jamil Mayet, Naveed Sattar, Paul M McKeigue, Nish Chaturvedi

**Affiliations:** 1 International Centre for Circulatory Health, National Heart and Lung Institute, Imperial College London, London, UK; 2 Division of Population Health Sciences and Education, St George’s University of London, London, UK; 3 National Heart and Lung Institute, Imperial College London, London, UK; 4 Institute of Cardiovascular and Medical Sciences, University of Glasgow School of Medicine, Glasgow, UK; 5 Centre for Population Health Sciences, University of Edinburgh, Edinburgh, UK; 6 International Centre for Circulatory Health, National Heart and Lung Institute, Imperial College London, London, UK

## Abstract

**Objective:**

To evaluate QRISK2 and Framingham cardiovascular disease (CVD) risk scores in a tri-ethnic UK population.

**Design:**

Cohort study.

**Setting:**

West London.

**Participants:**

Randomly selected from primary care lists. Follow-up data were available for 87% of traced participants, comprising 1866 white Europeans, 1377 South Asians, and 578 African Caribbeans, aged 40–69 years at baseline (1998–1991).

**Main outcome measures:**

First CVD events: myocardial infarction, coronary revascularisation, angina, transient ischaemic attack or stroke reported by participant, primary care or hospital records or death certificate.

**Results:**

During follow-up, 387 CVD events occurred in men (14%) and 78 in women (8%). Both scores underestimated risk in European and South Asian women (ratio of predicted to observed risk: European women: QRISK2: 0.73, Framingham: 0.73; South Asian women: QRISK2: 0.52, Framingham: 0.43). In African Caribbeans, Framingham over-predicted in men and women and QRISK2 over-predicted in women. Framingham classified 28% of participants as high risk, predicting 54% of all such events. QRISK2 classified 19% as high risk, predicting 42% of all such events. Both scores performed poorly in identifying high risk African Caribbeans; QRISK2 and Framingham identified as high risk only 10% and 24% of those who experienced events.

**Conclusions:**

Neither score performed consistently well in all ethnic groups. Further validation of QRISK2 in other multi-ethnic datasets, and better methods for identifying high risk African Caribbeans and South Asian women, are required.

## Introduction

Risk prediction is a cornerstone of strategies for prevention of cardiovascular disease (CVD).^1^ ^2^ The last 30 years have seen the derivation and modification of numerous risk calculators.^3^–^10^ People of South Asian origin experience greater risk than people of European origins, while in the UK, people of African Caribbean origin have lower risks of coronary heart disease (CHD), but higher risks of stroke.^11^ Earlier UK studies reported that the Framingham score (developed in a largely white US community) predicted no difference in risk between South Asians and white Europeans either with diabetes or in the general population in terms of CVD mortality^12^ and CHD and stroke risk.^13^ ^14^ The Framingham score has been criticised for lack of socioeconomic adjustment, overestimating risk in low risk and affluent populations, while underestimating risk in less affluent populations.^15^–^17^

More recently, QRISK2, including adjustment for deprivation and ethnicity, has undergone internal and external validation using UK primary care datasets.^4^ ^18^ However, the performance of QRISK2 in ethnic minorities was not reported separately.^18^ Earlier guidelines from the UK National Institute for Health and Care Excellence (NICE) recommended lower risk thresholds for South Asian men (but not women), by multiplying the Framingham risk scores by 1.4,^3^ ^19^ although this approach remains untested. In 2010 NICE recommended that the Framingham risk equation would no longer be recommended for CVD risk assessment, but that it could be considered together with other risk scores such as QRISK2. We evaluated the performance of Framingham^3^ ^19^) and QRISK2 scores as predictors of CVD outcomes over 10 years of follow-up in European, South Asian, and African Caribbean men and women in a UK population based cohort.

## Methods

SABRE (Southall And Brent REvisited) is a tri-ethnic, community based cohort from Southall and Brent (London).^20^ Participants aged 40–69 years at baseline (1988–1991) were randomly selected from primary care physician lists (n=4063) and workplaces (n=795). Ethnicity was agreed with the interviewer based on self-report, parental origins, and appearance. All South Asians and African Caribbeans were migrants. South Asians originated from the Indian subcontinent (India 90.3%, Pakistan 9.4%). Most African Caribbeans (92.5%) originated from the Caribbean and the remainder from West Africa.

At baseline, participants underwent fasting and post-glucose challenge blood tests, blood pressure measurements, ECG, anthropometry, and completed a health and lifestyle questionnaire.^20^ Minnesota criteria^21^ identified major Q waves on ECG. Atrial fibrillation and left ventricular hypertrophy (LVH) were identified in a subset of ECGs from European and African Caribbean participants.^20^ Diabetes was determined using WHO criteria^22^ or doctor diagnosed diabetes. Seated resting blood pressure was taken as the average of two readings measured using a random zero sphygmomanometer (Hawksley, UK).

Deaths were reported by the Office for National Statistics. During 2008–2011, survivors were invited to join a follow-up. This included a health and lifestyle questionnaire and/or primary care medical record review and/or attendance at our clinic at St Mary’s Hospital, London. Hospital episode statistics (HES) were obtained.

At follow-up we obtained data on family history of CHD, defined as angina or heart attack diagnosed in a parent aged under 60 years. We assigned Townsend 2001 deprivation scores based on output areas.^4^

### Identification of cardiovascular events during the first 10 years of follow-up

We mirrored end points for QRISK2 (first myocardial infarction, angina, CHD, stroke, transient ischaemic attack). We included coronary revascularisation procedures as these procedures incur a diagnosis of CHD on the general practice database.

For CHD, we identified the first event from any of the following sources:

- Cause of death includes International Classification of Disease (ICD) 9 codes 410-415 or ICD10 codes I200-I259.
- Primary care record review.
- Participant reported coronary revascularisation or acute myocardial infarction.
- HES: diagnostic ICD9 codes 410-415 or ICD10: I200-I259 or the Office of Populations and Surveys ‘Classification of interventions and procedures’: K401-K469, K491-504, K751-759 or U541.

For stroke/transient ischaemic attack, we identified the first event from any of the following sources:

- Cause of death includes ICD9 codes 430-439 or ICD10 codes I600-I698
- Primary care record review.
- HES: diagnostic ICD9 codes 430-439 or ICD10 codes: I600-I698.
- Participant reported physician diagnosed stroke (duration of symptoms >24 h).

Participants with CVD at baseline were excluded.

We performed sensitivity analyses using a stricter definition of CHD which excluded participant reported events and unconfirmed angina.

All participants gave written informed consent. Approval for the study at baseline was obtained from Ealing, Hounslow and Spelthorne, and University College London research ethics committees, and at follow-up from St Mary’s Hospital Research Ethics Committee (ref. 07/H0712/109).

### Statistical analyses

Ten year risks of CVD events were calculated using the Kaplan-Meier method. QRISK2 scores at baseline were calculated applying the published algorithm (http://svn.clinrisk.co.uk/qrisk2 XML source: Q68_qrisk2_2012_1_1.xml, STATA dta time stamp: 2 January 2012, 23:10). The Framingham risk score was calculated using the published algorithm^3^ with South Asian ethnicity adjustment.^19^ For primary analyses we assumed null values for baseline data which were not available for the majority of participants (see online supplemental table S1). We examined ethnicity specific calibration of each score by plotting observed against predicted risk by tenths of predicted risk and by calculation of the Brier score (lower values indicate greater accuracy) and the ratio of predicted to observed risk.

We assessed discrimination (differentiation of scores between participants who did and did not experience an event) by calculating the area under the receiver operating characteristics curve (AUROC) statistic for the end point of combined fatal and non-fatal CVD events. In addition, we calculated the D statistic (a measure of separation based on the ability of the prognostic index to discriminate between participants’ risks of an event) and R^2^ statistic,^23^ ^24^ which estimates the proportion of explained variance (higher values indicate better discrimination).

We compared high (≥20%) and low risk groups for each risk score and examined proportions of participants who would be reclassified to a different category using the alternative risk score and the proportion of observed events identified by high risk classification.

*Sensitivity analyses*—We repeated the above analyses recalculating QRISK2 and Framingham scores^19^ (a) using parental history data, (b) using the stricter definition of CHD, and (c) using the subset of African Caribbeans and Europeans with baseline ECG data for definition of LVH.

All analyses were conducted in STATA V.12.

## Results

Of the original 4539 participants without CVD at baseline, 4228 were traceable at follow-up. Follow-up data were available for 3821 (90%). Of measured risk factors, 89 (2.3%) participants had missing values for lipids, a Townsend score could not be assigned to 55 addresses (1.4%), and a further three had missing data for smoking or body mass index (BMI). Data on chronic kidney disease were not collected at baseline; however, only three participants had proteinuria and <5% had microalbuminuria (table 1). Only 1% of men and no women had atrial fibrillation at baseline (subset of 1163 European and African Caribbean participants). In the same subset, 13% had tall R waves on ECG, suggesting LVH. No participants were receiving statins at baseline. Assuming null values for family history, rheumatoid arthritis, atrial fibrillation, chronic kidney disease and LVH, we were able to calculate both QRISK2 and Framingham scores in 3674 (87% of those traced) (see online supplemental figure S1). Our study participants had higher Townsend scores (more deprived) than the general population of England and Wales (table 1). Baseline characteristics of those lost to follow-up or with missing baseline data were similar to those included in these analyses (see online supplemental Table S2).

Three-quarters of the participants were men, 49% were European, 36% were South Asian, and 15% were African Caribbean, reflecting the ethnicity–sex composition of the baseline group. As expected, diabetes was more frequent in South Asians and African Caribbeans. South Asians had less favourable lipid profiles, and African Caribbeans more favourable lipid profiles, than Europeans. Smoking was most frequent in Europeans (table 1).

**Figure 1:**
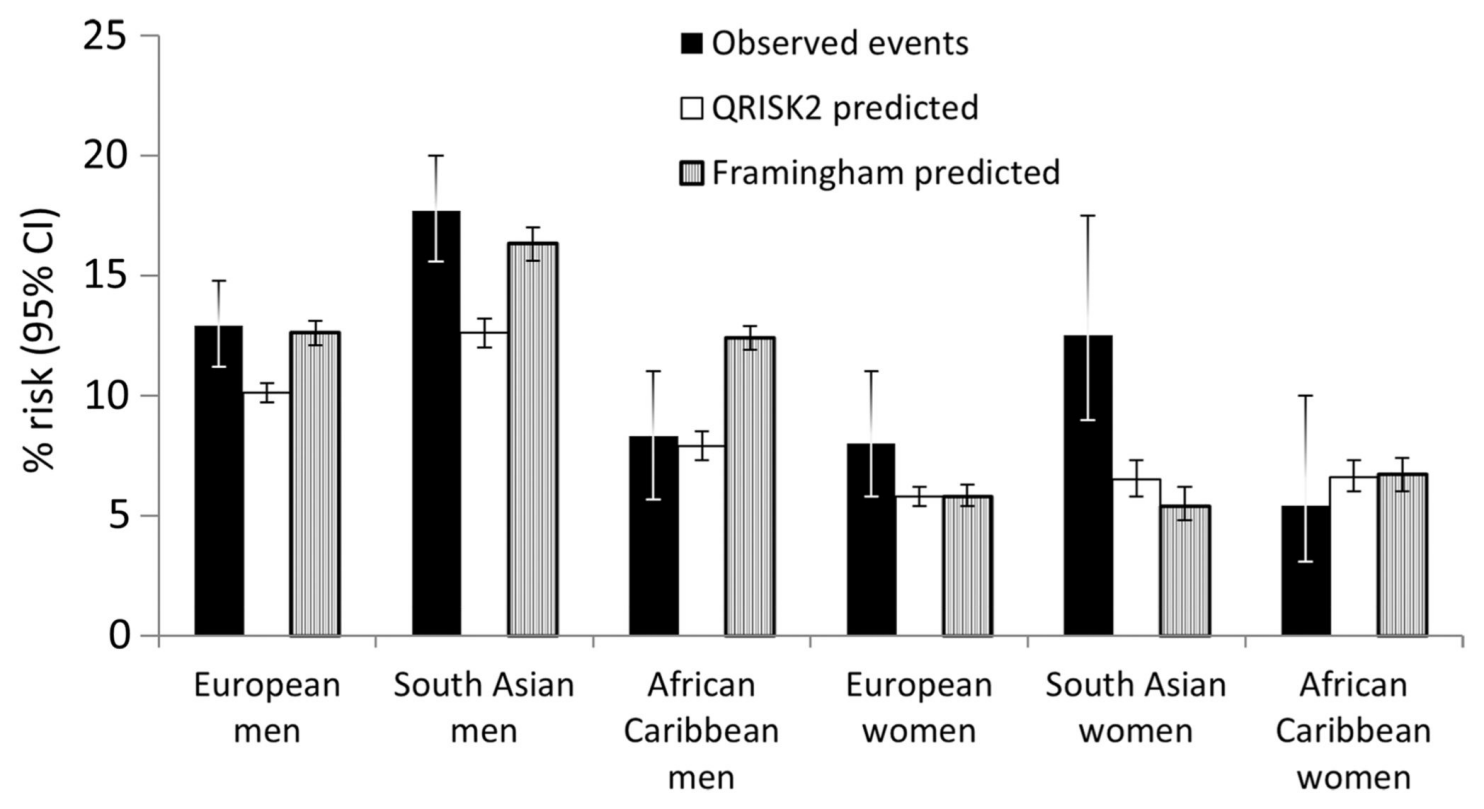
Observed and predicted risk over 10 years of follow-up.

During follow-up, 387 CVD events occurred in men (14%) and 78 in women (8%); 82% of these were CHD events. Rates were highest in South Asians and lowest in African Caribbeans (figure 1).

### Calibration

**Figure 2:**
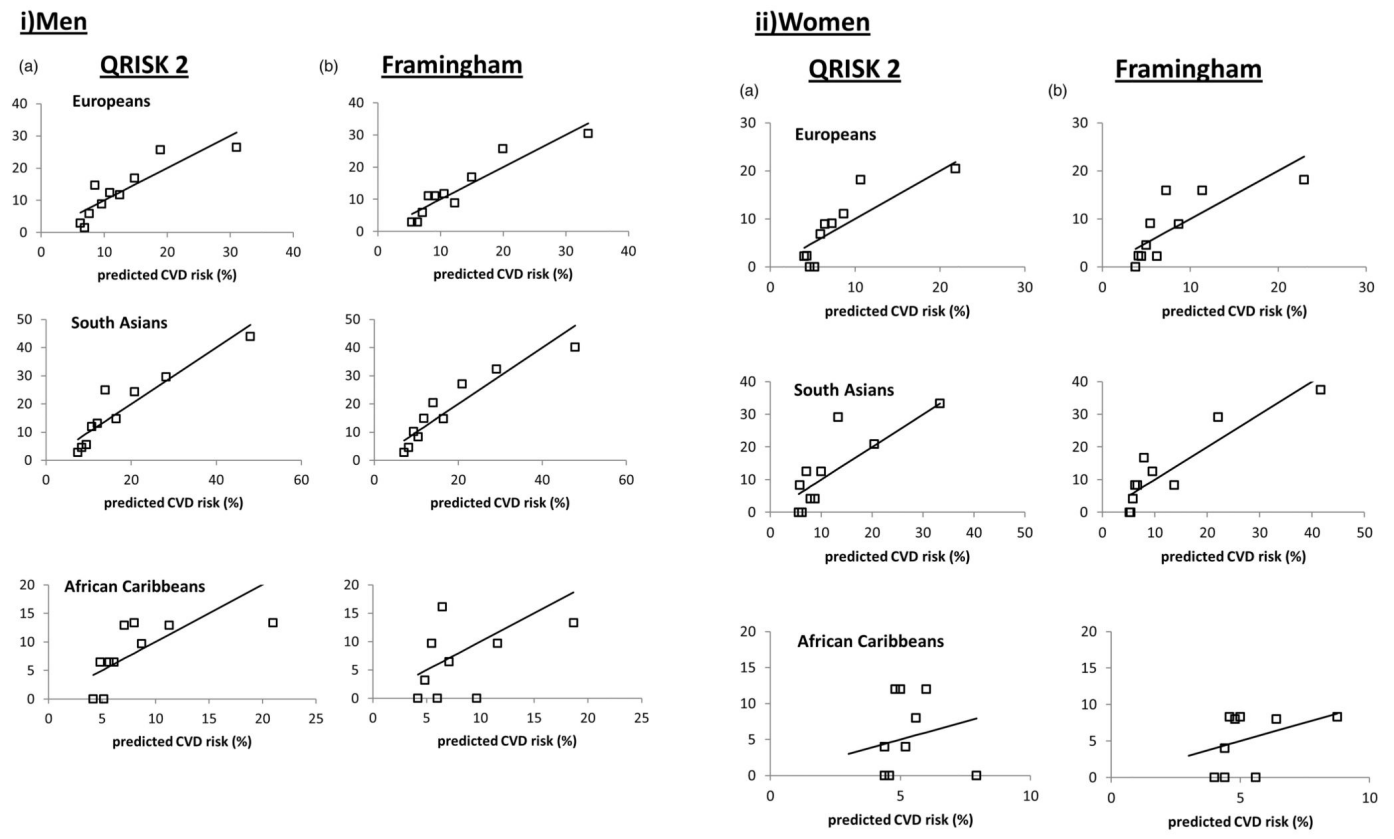
Plots of 10 year observed risk versus predicted risk of cardiovascular disease (CVD) (by tenths of predicted risk) for QRISK2 and Framingham (with South Asian male ethnicity adjustment) risk scores: (i) Men; (ii) Women.

Both scores under-predicted risk in European and South Asian women (ratio of predicted to observed risk: European women: QRISK2 0.73, Framingham 0.73; South Asian women: QRISK2 0.52, Framingham 0.43) (table 2). Both scores more closely approximated observed risk in European and South Asian men. In African Caribbean men, Framingham over-predicted, while QRISK2 showed a closer relationship with observed risk. In African Caribbean women both scores over-predicted; however, numbers of events were particularly small in African Caribbeans and Brier scores for both scores suggested better calibration in African Caribbeans (table 2, figures 1 and 2). In the subset of survivors with parental history data, both scores still notably under-predicted observed risk in South Asian women.

In the ECG subset, addition of LVH to the Framingham score increased over-prediction of risk in African Caribbeans and in European men.

### Discrimination

There was little difference in the discriminative performance of the two scores. The AUROC for men was 0.72 for both, and the D and R^2^ statistics were modest at 1.20% and 25.7% for QRISK2 and 1.22% and 26.2% for Framingham. In women, the D and R^2^ statistics were 1.31% and 29.1% for QRISK2 and 1.30% and 28.7% for Framingham. Discrimination was poorest for African Caribbeans for both scores (table 2). Repeat of discrimination analyses for the subset of survivors with family history data gave marginally better discrimination with overall AUROC of 0.74 for both scores. The D and R^2^ statistics in this subset overall were 1.30% and 28.7% (95% CI 21.0% to 36.0%) for QRISK2 and 1.34% and 30.0% (95% CI 22.3% to 37.3%) for Framingham. Addition of parental history data improved discrimination for both scores in African Caribbeans (AUROC: 0.75 for both scores). Addition of ECG identified LVH did not improve discrimination for Framingham.

### Classification

One third of men (925) were classified as high risk (≥20%) by Framingham compared with 617 (23%) men classified high risk by QRISK2. In women, 80 (9%) (Framingham) and 66 (7%) (QRISK2) were classified as high risk. In 683 men and women identified by QRISK2 as high risk, 193 (28%) had CVD events (accounting for 42% of total events). In 1025 men and women identified by Framingham as high risk, 251 (24%) had CVD events (accounting for 54% of total events). Reclassification from high risk Framingham to low risk QRISK2 would have occurred in 354 (38%) men and 29 (36%) women. Reclassification from high risk QRISK2 to low risk Framingham would have occurred in 46 (2.5%) men and 15 (1.7%) women (see online supplemental table S3). There were pronounced ethnic differences in classification. Of 107 African Caribbeans classified as high risk by Framingham, only nine experienced events (24% of total events), while of 38 African Caribbeans classified as high risk by QRISK2, only four experienced events (10% of total events). In 30 South Asian women who experienced events, QRISK2 identified 10 (33%) and Framingham identified 13 (43%) as high risk.

A similar picture was observed for classification in the subset with parental history data.

Further sensitivity analyses using a stricter definition of CHD in defining the CVD outcome produced similar findings for calibration, discrimination, and classification.

## Discussion

In this British population based cohort, QRISK2 underpredicted risk in South Asian and European men and women, while Framingham under-predicted risk in South Asian women and over-predicted in African Caribbeans. Both scores discriminated modestly between Europeans and South Asians who did and did not experience events, but performed less well in African Caribbeans. Using the conventional 20% threshold to identify people at high risk of CVD events, Framingham classified 50% more people as high risk than QRISK2. However, these high risk categories predicted only 54% (Framingham) and 42% (QRISK2) of all CVD events during 10 years of follow-up. Classification was particularly poor in African Caribbeans. Using these scores to define high risk African Caribbeans would predict less than one quarter of events. For South Asian women, QRISK2 high risk classification was also poor and would have predicted only one third of events. Inclusion of family history in risk score calculation improved discrimination (but not calibration) properties of both scores in African Caribbeans.

Given that the UK has large minority populations of South Asian and African Caribbean origins, with notably different rates of CVD compared with the European population, it is increasingly important that prevention measures are appropriately targeted.

We chose to consider the performance of QRISK2, which includes adjustments for ethnicity and deprivation and has been developed and tested using large UK primary care databases.^4^ ^18^ As a comparator, the Framingham 1991 score^3^ (incorporating NICE recommended adjustment for South Asian men), is familiar to most physicians and, until recently, was the risk predictor of choice according to UK national guidelines.^19^ We had expected QRISK2 to outperform the Framingham score in our UK tri-ethnic population, given that the Framingham score’s appropriateness to non-European populations with varied socioeconomic status has been questioned.^5^ ^16^ ^17^ However, our results do not suggest clear superiority of QRISK2 in men and women in any of the three ethnic groups. Underestimation of risk in South Asians, particularly in women, by both scores is of concern. The NICE ethnicity adjustment (Framingham score x 1.4) for South Asians has been recommended only for men,^19^ but our findings suggest that a risk multiplier for the Framingham score might also be considered for South Asian women and that further validation of QRISK2 is needed for this group. It is of note that the current Joint British Societies 2 guidelines do not advocate a South Asian multiplier, as it was considered that excess CHD risk in South Asians was explained by excess diabetes.^1^

For clinicians, classification to high risk categories is important in guiding implementation of preventive or therapeutic measures. The overall poor performance of the conventional cut-point of 20% in both risk scores in predicting events is worrying, as are the pronounced differences between the two risk scores in classification to high risk groups, particularly with regard to African Caribbeans and South Asian women.

We are not aware of other validation studies of QRISK2 in datasets beyond the QRESEARCH and THIN primary care datasets.^4^ ^18^ As is frequently observed, independent validation in different datasets may produce results less favourable than those of the original authors.^25^ This was the case for QRISK2 in our study which demonstrated poorer discrimination than has been reported in recent studies using primary care datasets.^4^ ^18^ Our own dataset, although small, contained few missing data, whereas the very large derivation and validation datasets had complete data for lipids, blood pressure, BMI, and smoking for only 18.4% and 19.6% of women and 16% and 19% of men, and used multiple imputation methods to overcome this.^8^ ^18^ Median follow-up was 6 years in the validation study, compared with our 10 year follow-up. These factors may contribute to the differences in performance of QRISK2 in our study compared with the validation studies. Surprisingly, the Framingham score, which predicted greater levels of risk in African Caribbeans, otherwise showed similar calibration and discrimination to QRISK2. However, our city dwelling study population was more deprived in terms of Townsend scores than the general population of England and Wales and, by design, included a large proportion of South Asians, who are known to be at high risk of CVD. Since the Framingham score does not include any socioeconomic adjustments, and has been reported to overpredict risk in comparison with QRISK2, it may serendipitously perform better in Europeans and South Asian men in this cohort, given previous reports of over-prediction of risk in more affluent populations and under-prediction in high risk groups.^16^ ^17^

A recent UK study compared QRISK2 and Framingham scores in association with national prevalence data in a UK black population and found, like us, that Framingham overestimated risk in black African Caribbeans, while QRISK2 performed better.^26^

### Strengths and limitations

To our knowledge this is the largest British multi-ethnic cohort with lengthy follow-up, extensive risk factors measured in mid-life, and only modest attrition for CVD outcomes. We did not have complete baseline data regarding LVH or atrial fibrillation, nor data on rheumatoid arthritis or chronic kidney disease. However, our data suggest that it is likely that only a few people in any ethnic group would have had chronic kidney disease at baseline and that <1% had atrial fibrillation. This is in keeping with data from derivation and validation studies for QRISK and QRISK2,^4^ ^18^ which showed that <1% had rheumatoid arthritis, and chronic kidney disease was present in <0.17%; hence, absence of these data is unlikely to affect our findings. Numbers of participants and CVD events are very small in women and in African Caribbean men and we urge caution in interpreting findings in these groups. Our main analyses assume null values for family history; however, findings in the subset of survivors who had parental history data collected at follow-up were similar to those observed in the main dataset. We also acknowledge that censoring due to non-CVD related deaths (n=86) may affect our findings. Our study baseline measurements were made over 20 years ago and the population characteristics for each ethnic group may have changed. We compared findings from the Health Survey for England’s (HSE) ethnic minority study in 2004, where, for example, the prevalence of diabetes in black Caribbean men was 5.3% in 35- to 54-year-olds and 24.8% in those aged 55+, which compares reasonably with prevalence of 17% in our group (mean age 53.5±5.8 years). For South Asian men, the corresponding HSE prevalences were 8.1% and 24.3% compared with 19% in our study group (mean age 50.8±6.9 years).^27^ It is also likely that our findings in first generation migrants may not be generalisable to future generations in each ethnic minority group.

## Conclusion

Over 10 years of follow-up in a UK population based cohort, QRISK2 and Framingham discriminated for CVD outcomes equivalently and modestly in European men and women and in South Asian men. Framingham over-predicted CVD events in African Caribbeans and both scores under-predicted in South Asian women. Classification to high risk groups differed notably between the two scores; neither high risk group performed well in predicting actual CVD events. Further validation of QRISK2 in other multi-ethnic datasets may be required. Particular attention should focus on identifying high risk African Caribbeans and South Asian women.

## Supplementary Material

► Additional material is published online only. To view please visit the journal online (http://dx.doi.org/10.1136/heartjnl-2013-304474).

Supplementary Figures and Tables

## Figures and Tables

**Table 1:** Baseline characteristics (means±SD, geometric means (95% CI) or n (%))

	European	South Asian	African Caribbean
*Men*			
N	1359	1076	307
Age	52.8±7.1	50.8±6.9	53.5±5.8
SBP, mm Hg	123±17	125±17	128±17
Total cholesterol, mmol/L	6.0 (5.9 to 6.0)	5.8 (5.8 to 5.9)	5.5 (5.4 to 5.6)
HDL cholesterol, mmol/L	1.3 (1.2 to 1.3)	1.2 (1.1 to 1.2)	1.4 (1.3 to 1.4)
Cholesterol: HDL ratio	4.7 (4.7 to 4.8)	5.0 (5.0 to 5.1)	3.9 (3.8 to 4.1)
BMI, kg/m2	26.2±3.9	25.9±3.3	26.4±3.4
Smoking			
Never	368 (27%)	811 (75%)	170 (55%)
Ex	537 (40%)	101 (9%)	58 (19%)
<10/day current	81 (6%)	53 (5%)	26 (8%)
10–19/day current	87 (6%)	65 (6%)	27 (9%)
20+/day current	3286 (21%)	46 (4%)	26 (8%)
Diabetes	81 (6%)	209 (19%)	53 (17%)
Treated hypertension	99 (7%)	136 (13%)	57 (19%)
Atrial fibrillation	2/210 (1%)	–	1/170 (0.6%)
Rheumatoid arthritis	n/a	n/a	n/a
Chronic kidney disease	n/a	n/a	n/a
Proteinuria (AER* ≥300 mg/day)	0/813	1/599 (0.02%)	0/205
Microalbuminuria (AER* ≥30 and <300 mg/day)	27/813 (3.3%)	15/599 (2.5%)	10/205 (4.9%)
LSOA based Townsend score (Deprivation index (quintiles for England and Wales))			
1 (most affluent)	65 (5%)	2 (0.2%)	0
2	44 (3%)	2 (0.2%)	2 (0.7%)
3	105 (8%)	24 (2%)	4 (1%)
4	620 (46%)	289 (27%)	75 (24%)
5 (least affluent)	525 (39%)	759 (71%)	226 (74%)
Townsend score	2.5 (2.3 to 2.6)	3.5 (3.4 to 3.7)	4.3 (4.0 to 4.6)
Family history of CHD	N=688	N=554	N=152
Parents diagnosed <60 years	66 (9.6%)	33 (6.0%)	3 (2.0%)
*Women*			
N	444	241	247
Age	53.0±6.8	50.3±6.5	52.6±6.0
SBP, mm Hg	119±16	124±20	131±17
Total cholesterol, mmol/L	6.0 (5.9 to 6.2)	5.7 (5.6 to 5.8)	5.5 (5.4 to 5.7)
HDL cholesterol, mmol/L	1.6 (1.6 to 1.7)	1.4 (1.3 to 1.4)	1.6 (1.6 to 1.7)
Cholesterol: HDL ratio	3.7 (3.6 to 3.8)	4.2 (4.0 to 4.3)	3.4 (3.3 to 3.5)
BMI, kg/m^2^	26.0±4.7	27.5±4.6	29.3±4.8
Smoking			
Never	212 (48%)	236 (98%)	206 (83%)
Ex	100 (23%)	1	19 (8%)
<10/day current	23 (5%)	3 (1%)	7 (3%)
10–19/day current	51 (11%)	0	10 (4%)
20+/day current	58 (13%)	1	5 (2%)
Diabetes	17 (4%)	38 (16%)	53 (21%)
Treated hypertension	46 (10%)	30 (12%)	69 (28%)
LSOA based Townsend score (Deprivation index (quintiles for England and Wales))			
1–2 (most affluent)	0	0	0
3	28 (6%)	1	3 (1%)
4	219 (49%)	101 (42%)	66 (26%)
5 (least affluent)	203 (45%)	143 (58%)	187 (73%)
Townsend score	3.3 (3.0 to 3.5)	3.3 (3.1 to 3.5)	4.9 (4.5 to 5.3)
Atrial fibrillation	0/249	–	0/238
Rheumatoid arthritis	n/a	n/a	n/a
Chronic kidney disease	n/a	n/a	n/a
Proteinuria (AER* ≥300 mg/day)	0/350	1/169 (0.06%)	0/189
Microalbuminuria (AER* ≥30 and <300 mg/day)	4/350 (1.1%)	4/169 (2.4%)	9/189 (4.8%)
Family history of CHD	N=214	N=118	N=128
Parents diagnosed <60 years	24 (11.2%)	14 (11.9%)	6 (4.7%)

* AER=albumin excretion rate from timed overnight urine collections, not available in all participants.

BMI, body mass index; CHD, coronary heart disease; HDL, high density lipoprotein; LSOA, lower layer super output area; SBP, systolic blood pressure.

**Table 2:** Discrimination and ratio of predicted to observed risk: QRISK2 and Framingham risk score by sex and ethnicity (95% CIs)

	QRISK2 score	Framingham CVD score
*Men*		
Europeans, n=1359		
AUROC	0.70 (0.66 to 0.74)	0.71 (0.67 to 0.75)
D statistic	1.06 (0.82 to 1.30)	1.13 (0.89 to 1.36)
R^2^ statistic %	21.1 (13.9 to 28.6)	23.3 (15.9 to 30.8)
Brier score	0.11 (0.10 to 0.13)	0.11 (0.10 to 0.12)
Predicted: observed	0.78 (0.72 to 0.85)	0.99 (0.96 to 1.00)
South Asians, n=1076		
AUROC	0.73 (0.69 to 0.77)	0.73 (0.69 to 0.77)
D statistic	1.22 (0.99 to 1.45)	1.23 (1.00 to 1.47)
R^2^ statistic %	26.3 (19.0 to 33.5)	26.6 (19.2 to 33.9)
Brier score	0.14 (0.12 to 0.15)	0.14 (0.12 to 0.15)
Predicted: observed	0.71 (0.64 to 0.78)	0.93 (0.88 to 0.96)
African Caribbeans, n=307		
AUROC	0.67 (0.57 to 0.77)	0.63 (0.53 to 0.73)
D statistic	0.96 (0.32 to 1.59)	0.80 (0.16 to 1.43)
R^2^ statistic %	17.9 (2.4 to 37.6)	13.2 (0.7 to 32.8)
Brier score	0.063 (0.040 to 0.085)	0.071 (0.052 to 0.091)
Predicted: observed	0.95 (0.80 to 1.00)	1.52 (1.24 to 2.06)
All, n=2742		
AUROC	0.72 (0.69 to 0.74)	0.72 (0.69 to 0.75)
D statistic	1.20 (1.04 to 1.36)	1.22 (1.06 to 1.38)
R^2^ statistic %	25.7 (20.6 to 30.8)	26.2 (21.1 to 31.3)
Brier score	0.12 (0.11 to 0.13)	0.12 (0.11 to 0.13)
Predicted: observed	0.75 (0.71 to 0.80)	0.99 (0.97 to 1.00)
*Women*		
Europeans, n=444		
AUROC	0.75 (0.67 to 0.82)	0.73 (0.65 to 0.80)
D statistic	1.33 (0.79 to 1.87)	1.29 (0.75 to 1.83)
R^2^ statistic %	29.7 (12.9 to 45.5)	28.5 (11.8 to 44.6)
Brier score	0.073 (0.046 to 0.10)	0.074 (0.053 to 0.095)
Predicted: observed	0.73 (0.57 to 0.88)	0.74 (0.57 to 0.88)
South Asians, n=241		
AUROC	0.75 (0.66 to 0.84)	0.77 (0.69 to 0.86)
D statistic	1.55 (0.91 to 2.19)	1.59 (0.96 to 2.21)
R^2^ statistic %	36.4 (16.6 to 53.3)	37.6 (18.1 to 53.9)
Brier score	0.10 (0.063 to 0.14)	0.10 (0.073 to 0.13)
Predicted: observed	0.52 (0.34 to 0.72)	0.43 (0.25 to 0.63)
African Caribbeans, n=247		
AUROC	0.65 (0.54 to 0.76)	0.62 (0.48 to 0.75)
D statistic	0.74 (0 to 1.63)	0.68 (0 to 1.58)
R^2^ statistic %	11.6 (0.1 to 38.9)	10.0 (0.04 to 37.2)
Brier score	0.066 (0.036 to 0.095)	0.066 (0.036 to 0.096)
Predicted: observed	1.22 (1.04 to 1.84)	1.24 (1.07 to 2.00)
All, n=932		
AUROC	0.73 (0.68 to 0.78)	0.72 (0.67 to 0.78)
D statistic	1.31 (0.94 to 1.68)	1.30 (0.93 to 1.67)
R^2^ statistic %	29.1 (17.5 to 40.2)	28.7 (17.1 to 39.9)
Brier score	0.078 (0.060 to 0.096)	0.079 (0.061 to 0.096)
Predicted: observed	0.74 (0.63 to 84)	0.70 (0.59 to 0.80)

AUROC, area under the receiver operating characteristics curve; CVD, cardiovascular disease.

## Key messages

**What is already known about this subject**

► Cardiovascular risk prediction is a cornerstone of preventive strategies.
► Until recently, UK national guidelines recommended the use of the Framingham score with an ethnicity related multiplier of 1.4 for South Asian men. More recently the QRISK2 risk score has been developed from UK primary care data and incorporates adjustments for ethnicity and socioeconomic position. QRISK2 has undergone internal and external validation in two general practice datasets, but its performance in ethnic minorities has not been reported separately.

**How might this impact on clinical practice**

► South Asians are at greatly elevated risk of both CHD and stroke, while in the UK African Caribbeans are at higher risk of stroke but lower risk of CHD compared with white Europeans. It is important that risk prediction scores are valid in all ethnic groups.

**What does this study add**

► Both Framingham and QRISK2 scores performed inconsistently across ethnic groups, particularly with regard to identifying high risk African Caribbeans and South Asian women in this British community based cohort. Further independent validation is needed in other multi-ethnic datasets and better methods of identifying high risk African Caribbeans and South Asian women are required.

## Appendix

*SABRE study group*:

Nish Chaturvedi (Imperial College London) (Principal Investigator)
Mark Baker (Imperial College London)
Norman Beauchamp (University of Washington, Seattle)
Emma Coady (Imperial College London)
Rory Collins (University of Oxford)
Nita Forouhi (Medical Research Council Epidemiology Unit, Cambridge)
Wladyslaw Gedroyc (Imperial College London)
Ian Godsland (Imperial College London)
Andrew Hattersley (Peninsula Medical School, University of Exeter)
John Heasman (Imperial College London)
Alun Hughes (Imperial College London)
Daniel Key (Imperial College London)
Azeem Majeed (Imperial College London)
Katherine March (Imperial College London)
Jamil Mayet (Imperial College London)
April McGowan (Imperial College London)
Paul McKeigue (University of Edinburgh)
Chloe Page (Imperial College London)
Martin Prince (Kings College London)
Marcus Richards (Medical Research Council, Unit for Lifelong Health and Ageing)
Naveed Sattar (University of Glasgow)
Dean Shibata (University of Washington, Seattle)
Robert Stewart (Kings College London)
Therese Tillin (Imperial College London)
Claire Tuson (Imperial College London)
Peter Whincup (St George’s, University of London)
Joe Willis (Imperial College London)
Andrew Wright (Imperial College London)
