# Supplementary material for: Ethnicity and prediction of cardiovascular disease: performance of QRISK2 and Framingham scores in a UK tri-ethnic prospective cohort study (SABRE—Southall And Brent REvisited)
Source: Heart. Author manuscript; Available in PMC 2016 May 17. (PMC4869829; doi:10.1136/heartjnl-2013-304474)

Supplemental tables/figures

Table S1:Baseline variables included in calculation of QRISK2 and Framingham scores

	QRISK 2	Framingham
Ethnicity (European, Indian Pakistani, Bangladeshi, Black African, Black Caribbean)	√	√ For South Asian men score is multiplied by 1.4
Age, years	√	√
Sex	√	√
Smoking: Current or non-smoker (includes ex-smoker)	√	√
Systolic blood pressure	√	√
Total cholesterol: high density lipoprotein ratio	√	√
Type 2 diabetes	√	√
Body mass index	√	Not applicable
Treated hypertension	√	Not applicable
Townsend deprivation score (output area level 2001 census data)	√	Not applicable
Family history of CHD in first degree relative under 60. *Available only from survivors at 20 year follow-up: history of CHD in parents diagnosed at <60years)*	Sensitivity analyses only. Otherwise null values assumed.	Sensitivity analyses only. (multiply score by 1.5 if any positive family history) Otherwise null values assumed.
Rheumatoid arthritis. Not available.	Null values assumed	Not applicable
Chronic renal disease. Not available.	Null values assumed	Not applicable
Atrial fibrillation. Available only in subset.	Null values assumed.	Not applicable
Left ventricular hypertrophy. Available only in subset.	Not applicable	Sensitivity analyses only. Otherwise null values assumed.

Table S2. Baseline characteristics of those lost to follow-up or excluded from analyses due to missing baseline data (means±SD, geometric means(95% CIs)

Men	European	Indian Asian	African Caribbean
N	428	343	146
Age	53.3±7.3	52.0±7.2	54.2±5.9
SBP, mm Hg	122±17	127±18	130±19
HDL cholesterol	1.21(1.17, 1.24)	1.14(1.11, 1.18)	1.44(1.37, 1.52)
Cholesterol: HDL ratio	4.95(4.77, 5.13)	5.24(5.07, 5.42)	3.84(3.61, 4.08)
Body mass index	26.4±3.8	25.9±3.3	26.2±3.3
Smoking:			
Never	105(25%)	226(66%)	67(47%)
Ex	176(41%)	52(18%)	30(21%)
Current	146(34%)	62(15%)	46(32%)
Diabetes	44(10%)	99(29%)	29(20%)
Treated hypertension	51(12%)	54(16%)	28(19%)
Townsend score	2.5(2.3, 2.8)	2.3(1.8, 3.1)	4.3(3.9, 4.7)

Women			
N	115	50	101
Age	53.6±6.4	53.0±7.8	53.9±5.6
SBP, mm Hg	121±17	129±23	131±16
HDL cholesterol	1.53(1.46, 1.61)	1.35(1.24, 1.46)	1.64(1.55, 1.73)
Cholesterol: HDL ratio	3.86(3.64, 4.10)	4.15(3.83, 4.49)	3.40(3.18, 3.64)
Body mass index	26.1±5.0	26.1±4.2	29.6±4.9
Smoking:			
Never	55(48%)	46(96%)	83(84%)
Ex	25(22%)		10(10%)
Current	35(30%)	2(4%)	6(6%)
Diabetes	7(6%)	9(18%)	16(16%)
Treated hypertension	13(11%)	8(16%)	33(33%)
Townsend score	3.4(2.9, 3.9)	3.6(3.2, 4.2)	5.3(4.7, 5.8)

Supplemental Table S3: Comparison of QRISK2 and Framingham scores in classification into low and high 10 year risk of cardiovascular events and observed and predicted risk

	QRISK2		Total
**MEN**			
Framingham score (with South Asian ethnicity adjustment): **low risk (<=20%)**	Low risk (<20%)	High risk(>=20%)	
Number of men<br>Number of events (10 year risk (95%CI)%)<br>QRISK2: geometric means(95% CI)%<br>Framingham: geometric means(95% CI)%	1771<br>149(8.4(7.2, 9.8))<br>7.1(6.9, 7.3)<br>9.3(9.1, 9.5)	46 (2.5%)<br>7(15.4(7.6, 29.6))<br>22.3(21.7, 22.9)<br>16.7(15.9, 17.5)	1817<br>156(8.7(7.4, 10.0))<br>7.3(7.1, 7.5)<br>9.43(9.2, 9.7)
Framingham (with South Asian ethnicity adjustment): **high risk (>20%)**			
Number of men<br>Number of events(10 year risk (95%CI)%)<br>QRISK2: geometric means(95% CI)%<br>Framingham: geometric means(95% CI)%	354(38.3%)<br>58(16.6(13.1, 20.9))<br>15.2(14.8, 15.6)<br>23.9(23.5, 24.3)	571<br>173(32.1(27.4, 35.2))<br>29.3(28.7, 30.0)<br>34.3(33.5, 35.2)	925<br>231(25.5(22.8, 28.5))<br>22.80(22.21, 23.41)<br>29.9(29.3, 30.5)
**WOMEN**			
Framingham score: **low risk (<=20%)**			
Number of women<br>Number of events(10 year risk (95%CI)%)<br>QRISK2: geometric means(95% CI)%<br>Framingham: geometric means(95% CI)%	837<br>58(6.9(5.4, 8.8))<br>5.4(5.1, 5.6)<br>5.1(4.8, 5.3)	15(1.7%)<br>0<br>22.8(21.2, 24.5)<br>15.5(13.2, 18.2)	852<br>58(6.9(5.4, 8.8))<br>5.5(5.2, 5.8)<br>5.2(4.9, 5.5)
Framingham): **high risk (>20%)**			
Number of women<br>Number of events(10 year risk(95%)%)<br>QRISK2: geometric means(95% CI)%<br>Framingham: geometric means(95% CI)%	29 (36.3%)<br>7(24.2(22.3, 44.1)<br>16.2(15.0, 17.4)<br>22.5(21.6, 23.5)	51<br>13(26.4(16.3, 41.1)<br>28.1(26.2, 30.2)<br>28.7(26.9, 30.6)	80<br>20(25.6(17.4, 35.6))<br>23.00(21.26, 24.87)<br>26.3(25.0, 27.7)

Figure S1:

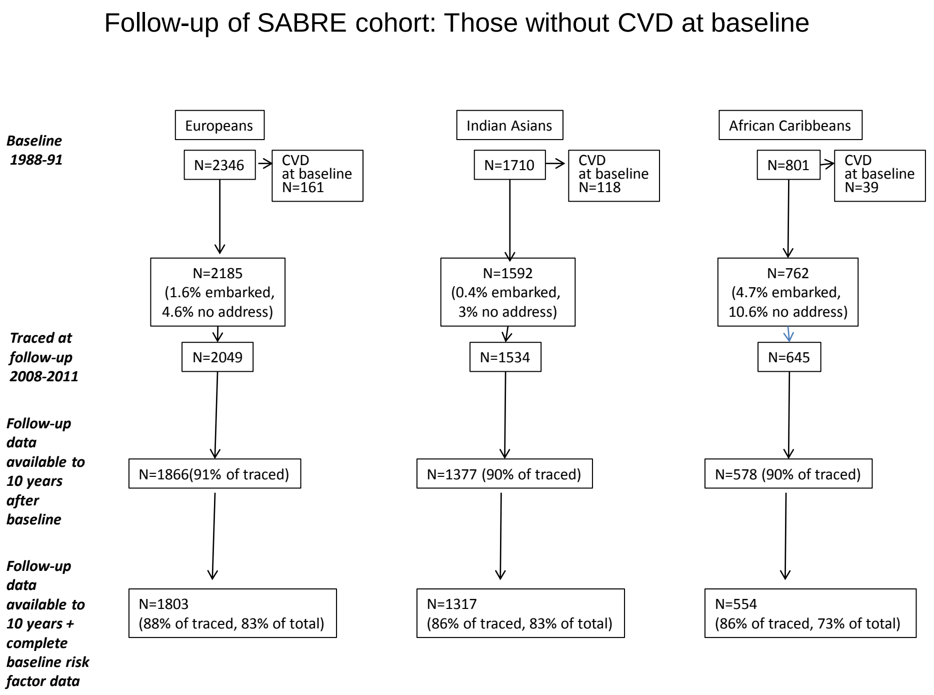
Follow-up of SABRE cohort: Those without CVD at baseline
Baseline
1988-91
Europeans
N=2346
CVD at baseline N=161
N=2185 (1.6% embarked, 4.6% no address)
N=2049
N=1866(91% of traced)
N=1803 (88% of traced, 83% of total)
Indian Asians
N=1710
CVD at baseline N=118
N=1592 (0.4% embarked, 3% no address)
N=1534
N=1377 (90% of traced)
N=1317 (86% of traced, 83% of total)
African Caribbeans
N=801
CVD at baseline N=39
N=762 (4.7% embarked, 10.6% no address)
N=645
N=578 (90% of traced)
N=554 (86% of traced, 73% of total)
Traced at follow-up 2008-2011
Follow-up data available to 10 years after baseline
Follow-up data available to 10 years + complete baseline risk factor data

Figure S2) : Plots of 10 year predicted vs observed risk ratios (95% CIs), by tenths of predicted risk, for QRISK2 and Framingham (with South Asian male ethnicity adjustment) risk scores

a)Men

i)QRISK 2 ii)Framingham

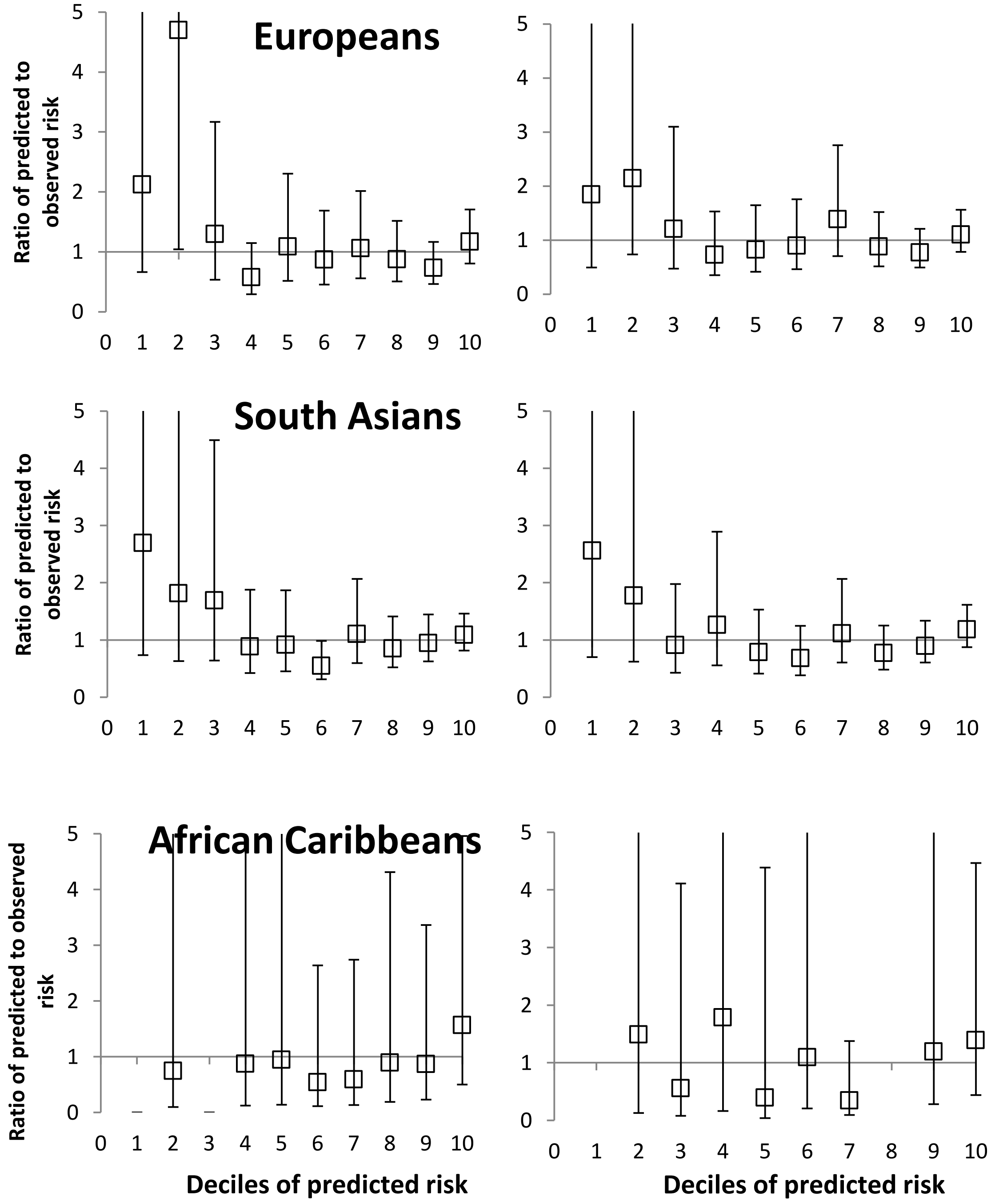

Figure S2(continued)

b)Women

i)QRISK 2 ii) Framingham

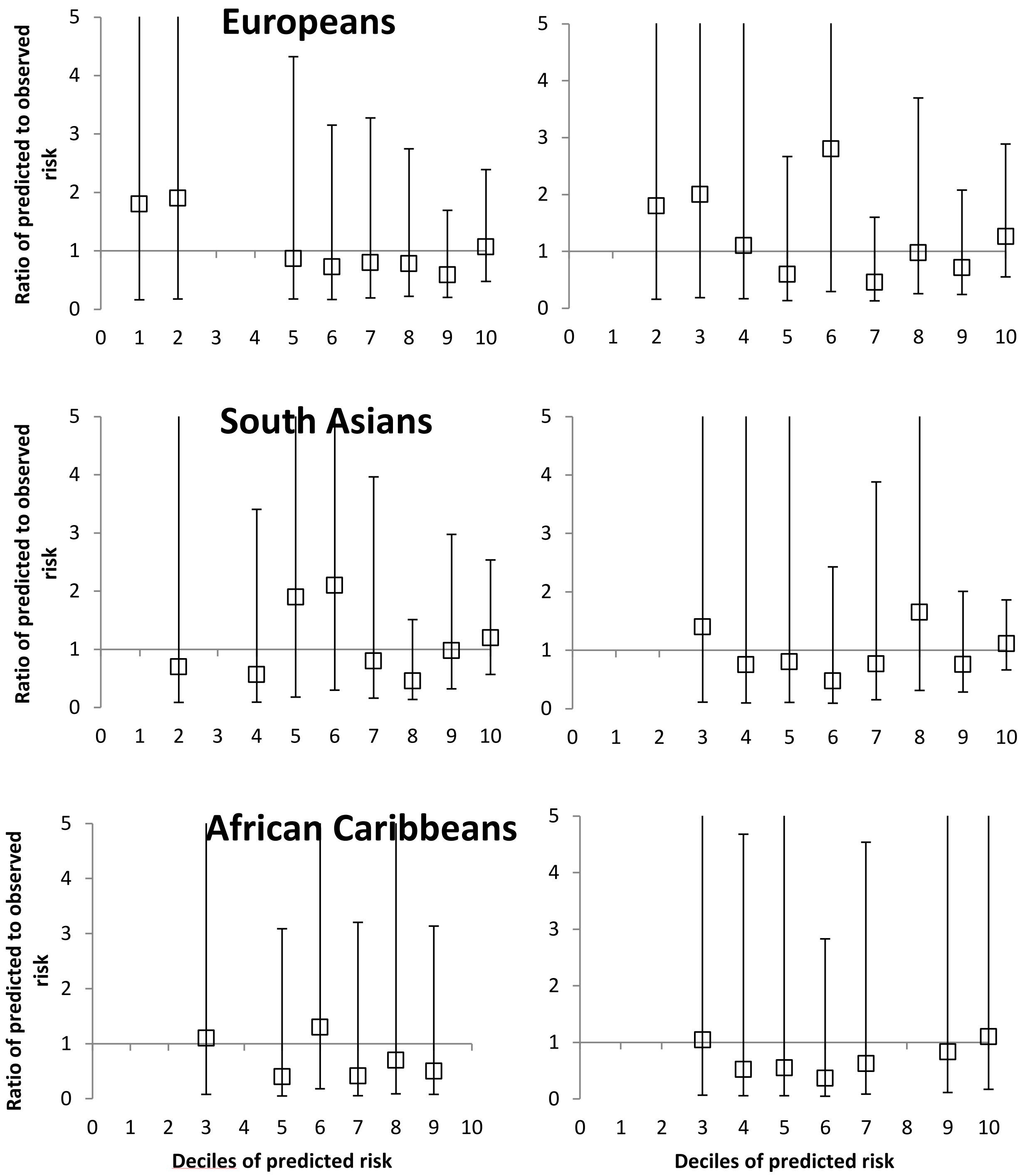

Supplement: Supplementary Figures and Tables [file NIHMS67981-supplement-Supplementary_Figures_and_Tables.pdf]
